# County Poverty Concentration and Disparities in Unintentional Injury Deaths: A Fourteen-Year Analysis of 1.6 Million U.S. Fatalities

**DOI:** 10.1371/journal.pone.0153516

**Published:** 2016-05-04

**Authors:** Rebecca A. Karb, S. V. Subramanian, Eric W. Fleegler

**Affiliations:** 1 Alpert Medical School at Brown University, Department of Emergency Medicine, Providence, Rhode Island, United States of America; 2 Harvard T.H. Chan School of Public Health, Boston, Massachusetts, United States of America; 3 Boston Children’s Hospital, Division of Emergency Medicine, Boston, Massachusetts, United States of America; Public Health Agency of Canada, CANADA

## Abstract

Unintentional injury is the fourth leading cause of death in the United States, and mortality due to injury has risen over the past decade. The social determinants behind these rising trends have not been well documented. This study examines the relationship between county-level poverty and unintentional injury mortality in the United States from 1999–2012. Complete annual compressed mortality and population data for 1999–2012 were obtained from the National Center for Health Statistics and linked with census yearly county poverty measures. The outcomes examined were unintentional injury fatalities, overall and by six specific mechanisms: motor vehicle collisions, falls, accidental discharge of firearms, drowning, exposure to smoke or fire, and unintentional poisoning. Age-adjusted mortality rates and time trends for county poverty categories were calculated, and multivariate negative binomial regression was used to determine changes over time in both the relative risk of living in high poverty concentration areas and the population attributable fraction. Age-adjusted mortality rates for counties with > 20% poverty were 66% higher mortality in 1999 compared with counties with < 5% poverty (45.25 vs. 27.24 per 100,000; 95% CI for rate difference 15.57,20.46), and that gap widened in 2012 to 79% (44.54 vs. 24.93; 95% CI for rate difference 17.13,22.09). The relative risk of living in the highest poverty counties has increased for all injury mechanisms with the exception of accidental discharge of firearms. The population attributable fraction for all unintentional injuries rose from 0.22 (95% CI 0.13,0.30) in 1999 to 0.35 (95% CI 0.22,0.45) in 2012. This is the first study that uses comprehensive mortality data to document the associations between county poverty and injury mortality rates for the entire US population over a 14 year period. This study suggests that injury reduction interventions should focus on areas of high or increasing poverty.

## Introduction

Unintentional injury is the fourth leading cause of death in the United States (US)—and the leading cause for individuals under 45 years old—accounting for over 130,000 deaths in 2013[1]. Injuries are responsible for roughly as many deaths each year as stroke or acute myocardial infarction[2]. Moreover, in contrast to mortality from most diseases, rates of injury mortality in the US have risen over the past decade[3–5]. The social determinants behind these trends are not well understood.

Neighborhood poverty has consistently been associated with a range of individual health outcomes, including overall mortality and life expectancy[6,7], cardiovascular disease[8], low birth weight[9], asthma admission rates[10], and obesity and diabetes[11]. There is a growing appreciation of the role of the social and physical neighborhood environment in differentially shaping individuals’ access to health services, influencing health behavior through the built environment, and patterning exposure to violence, stressors, and potentially deleterious social norms[12,13]. In contrast to other health outcomes and behaviors, the relationship between area disadvantage and injury has received relatively little attention.

The poverty rate in the US steadily rose during the first decade of the century, climbing from 12.2% in 2000 to 15.3% in 2010[14], and has remained relatively stable for the past 5 years. In addition to the negative impact on the individuals themselves who have become impoverished, this increase also changes the social and physical environment to which “non-poor” residents are exposed. In 1999, about 5% of the US population lived in counties that had a greater than 20% poverty rate; by 2012, over 15% of the population lived in such counties. As a result, more individuals have become exposed to the potentially detrimental effects of concentrated neighborhood poverty.

The aim of this study is to examine the association between county-level poverty and unintentional injury mortality in the US from 1999–2012, overall and by six specific mechanisms. Associations between area socioeconomic status and injury rates have been observed in other countries[15,16], for particular age groups[17,18], and in site-specific studies[19,20]. To the authors’ knowledge, this is the first study that uses comprehensive mortality data to document the associations between county poverty and injury mortality rates for the entire US population.

## Study Design and Data

### Patients

No patients were involved in setting the research question or the outcome measures, nor were they involved in the design and implementation of the study. There are no plans to involve patients in the dissemination of results as the nature of the data precludes individual patient identification.

### Data

Annual compressed mortality and population data for 1999–2012 were obtained from the National Center for Health Statistics (NCHS)[21]. The data provides complete pooled annual mortality and population counts by age, sex, race/ethnicity, and cause of death at the county level. Cause of death is coded in accordance with the International Statistical Classification of Diseases and Related Health Problems, 10th Revision (ICD-10). Population counts are based on bridged-race revised intercensal estimates. We examined combined total unintentional injury mortality, as well as six specific causes of mortality due to injury: motor vehicle collisions (MVC), falls, accidental discharge of firearms, drowning, exposure to smoke or fire, and unintentional poisoning. For the denominator, the mortality file was linked with Census population data for age, sex, and race/ethnicity categories at the county level.

County poverty rate estimates were obtained through the Census from the Small Area Income and Poverty Estimates website. NCHS 2006 urban-rural county classification was obtained from the CDC and is based on the 2000 census population. County poverty rates, the percentage of the population living below the federal poverty level ($16,500 for a family of 4 in 1999, $22,050 in 2010), were divided into 5 groups, less than 5% living in poverty, 5 to <10%, 10 to <15%, 15 to <20% and 20% or more living in poverty, the federal definition of a “poverty area”[22].

### Statistical Analyses

Age was coded as a categorical variable, with the following categories: 0–14, 15–24, 25–44, 45–64, 65–84, and 85 years and older. Race/ethnicity was constructed as a four category variable: white, black, Hispanic, and other (includes Asians and American Indians). For descriptive analyses of trends over time in injury mortality within county poverty categories, we aggregated mortality counts to the county poverty category level using methods outlined in The Public Health Disparities Geocoding Project[23]. We calculated annual age-standardized (with the 2000 population as referent) mortality rates for each county poverty category using age categories and the direct method[24]. The CDC mortality file consists of population data; therefore, it is not subject to sampling error.

For multivariate analyses, we aggregated mortality counts by unique cross tabulation of year, age, race/ethnicity, sex, county poverty category, and county urban/rural classification, yielding 20,160 total observations. Aggregation to this level reduces the level of error for both the numerator (number of deaths) and denominator (population estimates). We performed negative binomial regression due to overdispersion, with the outcome being counts per cell, to calculate the relative risk (RR) for total unintentional injury mortality as well as the six specific causes.

The population attributable fraction (PAF) was calculated using the adjusted RRs from year-specific negative binomial regressions, and takes into account the multiple levels of exposure of the county poverty variable. The PAF represents the proportion of total deaths that are associated with county poverty, or, conversely, the reduction in the number of deaths that could potentially be seen if the exposure were eliminated (i.e. if all counties had poverty rates less than 5%). The PAF was calculated using the following formula to account for multiple levels of the exposure variable [25]. Adjusted RR from the multivariate negative binomial regressions (adjusted for age, sex, race, and urban status) were used to minimize confounding.

$$PAF=\frac{\left(p_{0}+(p_{1}*{RR}_{1})+(p_{2}*{RR}_{2})\ldots (p_{n}*{RR}_{n})\right)-1}{p_{0}+(p_{1}*{RR}_{1})+(p_{2}*{RR}_{2})\ldots (p_{n}*{RR}_{n})}$$

For falls, the PAF for county poverty was negative and so the Preventative Fraction (PF) was calculated in its place (1-PF = 1/[1-PAF]). The PF represents the proportion reduction in deaths that may be seen if all individuals were exposed to the “protective factor”. CIs for the PAF were derived using the substitution method[26] and are reported in **S3 Table.**

## Results

**Table 1** reports mortality rates (per 100,000) for all unintentional injuries combined, as well as the six specific causes, for the years 1999 and 2012. Overall age-adjusted mortality from unintentional injuries increased 7% over the 14 year period, from 35.23 to 38.64 per 100,000 (Δ 3.41; 95% CI 3.11,3.72). The greatest increases were seen among whites (Δ 8.08; 95% CI 7.70,8.46) and individuals 45–64 years (Δ12.36; 95% CI 11.72,12.99), with decreases among blacks (-Δ8.18; 95% CI

**Table 1:** Mortality Rates (per 100,000)^a^ for All Unintentional Injuries and Specific Causes, US 1999–2012.

							Specific Causes							
	All Unintentional Injuries		MVC		Falls		Accidental Discharge of Firearms		Drowning		Fire/Smoke Exposure		Poisoning	
	1999	2012	1999	2012	1999	2012	1999	2012	1999	2012	1999	2012	1999	2012
**Age**														
0-14	**9.73**	**6.66**	4.34	2.21	0.20	0.09	0.15	0.09	1.55	1.16	1.01	0.37	0.14	0.15
15-24	**35.31**	**27.11**	26.19	16.07	0.63	0.50	0.65	0.27	1.67	1.23	0.52	0.19	2.49	7.23
25-44	**31.81**	**37.29**	15.85	13.20	1.16	0.94	0.33	0.19	1.08	0.97	0.82	0.40	8.10	18.88
45-64	**31.35**	**43.71**	13.82	12.40	2.83	4.24	0.23	0.18	0.98	1.18	1.14	1.01	5.82	19.03
65-84	**66.87**	**66.72**	21.27	14.74	18.01	29.81	0.18	0.17	1.12	1.18	2.95	2.06	1.84	3.80
>85	**282.35**	**337.27**	30.36	22.10	110.21	222.42	0.24	0.07	1.64	1.45	5.90	3.64	3.44	4.05
**Race/Ethnicity**														
White	**35.48**	**43.56**	15.44	12.09	5.01	8.86	0.30	0.19	1.14	1.13	1.06	0.74	4.19	14.29
Black	**40.77**	**32.59**	16.29	11.86	3.44	3.70	0.43	0.25	1.71	1.41	3.01	1.32	6.38	8.36
Hispanic	**30.26**	**26.24**	14.27	9.64	3.92	5.66	0.18	0.09	1.20	0.68	0.69	0.41	4.78	6.16
Other	**24.50**	**22.18**	12.35	7.23	3.70	5.39	0.11	0.06	1.35	1.33	0.64	0.39	1.68	4.42
**Sex**														
Male	**49.51**	**52.06**	21.26	16.46	6.50	9.85	0.51	0.31	2.01	1.77	1.58	0.94	6.43	14.83
Female	**22.41**	**26.12**	9.59	6.50	3.57	6.66	0.08	0.05	0.51	0.49	0.89	0.56	2.30	7.96
**Urban/Rural**^**b**^														
Large Central Metro	**30.51**	**32.31**	10.51	7.81	4.96	7.69	0.17	0.08	1.11	0.96	1.04	0.52	6.37	10.98
Large Fringe metro	**29.57**	**34.66**	12.66	9.39	4.49	8.03	0.17	0.10	1.00	0.94	0.85	0.49	3.22	10.80
Medium metro	**35.19**	**40.04**	14.97	11.31	4.89	8.55	0.27	0.19	1.29	1.23	1.24	0.69	4.29	12.23
Small metro	**38.48**	**41.80**	17.78	13.12	5.06	7.99	0.34	0.28	1.46	1.21	1.44	1.00	3.13	11.13
Micopolitan	**44.86**	**49.77**	22.47	17.71	4.67	8.25	0.57	0.34	1.55	1.48	1.66	1.14	3.10	12.62
Rural	**55.10**	**58.56**	30.36	24.37	4.88	8.03	0.86	0.41	1.95	1.69	2.00	1.72	2.97	12.61
**County Poverty**														
0-<5%	**27.24**	**24.93**	10.38	5.47	5.54	6.34	0.11	0.04	0.74	0.56	0.47	0.26	2.65	7.03
5-<10%	**30.78**	**32.79**	12.79	8.43	4.96	8.54	0.14	0.09	1.06	0.84	0.86	0.44	3.48	9.40
10-<15%	**36.91**	**37.62**	15.79	10.40	4.87	8.44	0.34	0.15	1.35	1.10	1.35	0.60	4.65	11.36
15-<20%	**38.40**	**39.04**	17.27	11.42	4.44	8.11	0.40	0.16	1.38	1.15	1.50	0.73	5.21	11.80
≥20%	**45.25**	**44.54**	21.77	15.27	4.38	6.94	0.59	0.31	1.64	1.32	1.81	1.24	5.89	12.35
**Total**	**35.23**	**38.64**	**15.20**	**11.37**	**4.81**	**8.06**	**0.30**	**0.17**	**1.26**	**1.12**	**1.20**	**0.73**	**4.34**	**11.37**

Source: National Center for Health Statistics (Compressed Mortality File 1999–2010)

^a^ Rates for Race/Ethnicity, Sex, County Poverty and Urban/Rural Classification are age-standardized to 2000 population

^b^ The 2006 NCHS urban-rural classification scheme for counties is based on the 2000 census, and includes 6 categories: large central metropolitan (central counties of MSAs of greater than 1 million population), large fringe metropolitan (fringe counties of MSAs of greater than 1 millions population), medium metropolitan (counties within MSAs of 250,000–999,999 population), small metropolition (counties within MSAs of 50,000–249,999 population), micropolitan (counties with an urban core of 10,000 to 49,999 population), and rural (counties with no urban core).

-7.24,-9.11), Hispanics (-Δ4.02; 95% CI -3.12,-4.93), and young adults 15–24 (-Δ8.2; 95% CI -7.44,-8.97). The increase in overall injury mortality appears to be driven primarily by increases in falls (Δ 3.25; 95% CI 3.12,3.38) and unintentional poisonings (Δ7.03; 95% CI 6.89,7.17). These increases were offset predominantly by a decline in mortality due to motor vehicle collisions (-Δ3.83; 95% CI -3.64,-4.01) and fire/smoke exposure (Δ-0.47; 95% CI -0.43;-0.51). Mortality rates for falls and poisoning have approached rates of death from motor vehicle collisions.

Overall injury mortality rates are highest for individuals over 85, owing mostly to high rates of death from falls, which by 2012 accounted for 66% of injury mortality for this age group. From 1999–2012, injury rates among children and youth significantly declined, while rates for other age groups increased. The largest percentage increase among the 45–64 age group was driven primarily by the large increase in unintentional poisoning (Δ13.21; 95% CI 12.86,13.57) in this age group.

Injury mortality rates for whites have risen since 1999, and have surpassed injury mortality rates for blacks. In 2012, whites had a mortality rate of 43.56 compared to 32.59 for blacks, 26.24 for Hispanics, and 22.18 for other racial/ethnic groups. Once again, this jump among whites was driven by disproportionate increases in rates of falls and poisonings.

Age-adjusted mortality rates by county poverty concentration category show that counties with greater than 20% poverty had a 66% higher rate of injury mortality in 1999 than counties with less than 5% poverty (45.25 vs. 27.24 per 100,000; 95% CI for rate difference 15.57,20.46). The gap between counties widened in 2012 to 79% (44.54 vs. 24.93 per 100,000; 95% CI for rate difference 17.13,22.09) due to decreases in mortality within low poverty areas. With the exception of falls, mortality rates were dramatically higher among high poverty counties compared to low poverty counties across all mechanisms.

**Fig 1:**
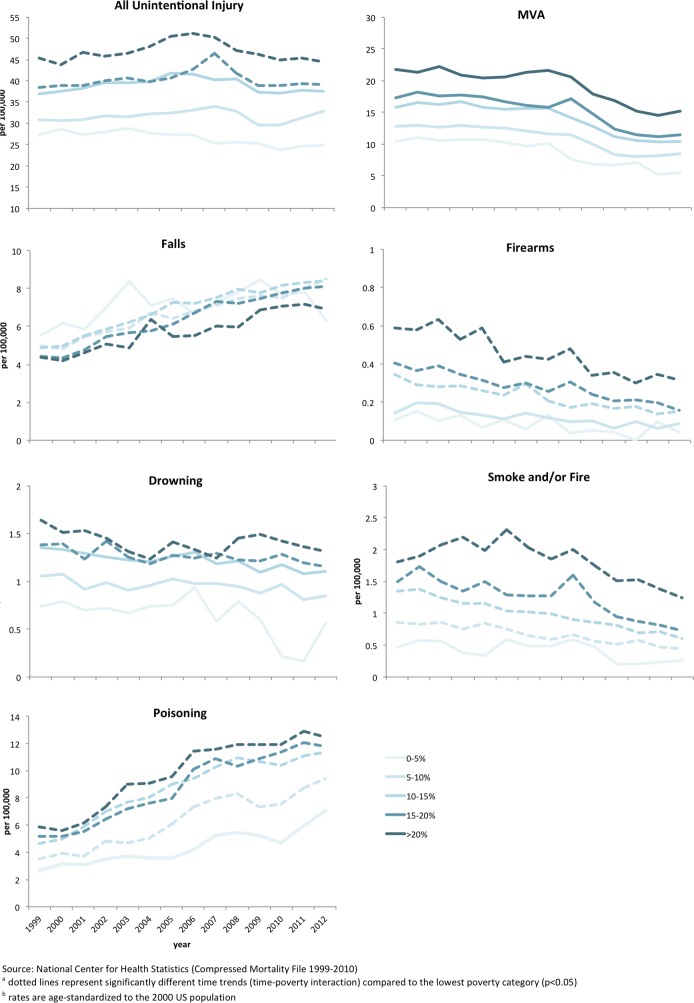
Trends in Mortality for All Unintential Injuries and Specific Causes by County Poverty Rates, US 1999–2012.

**Fig 1** shows time trends for age-standardized rates by county poverty category for all unintentional injuries and the six specific injury mechanisms. There is a significant and increasing disparity for overall injury mortality between low and high poverty counties from 1999 to 2012, with declining rates found for the lowest poverty counties and steady rates among high poverty counties. In addition, a time-poverty interaction was significant for falls, poisoning, and drownings, indicating more rapidly increasing rates within higher poverty areas. The most striking trend was among poisoning deaths, with greater increases seen within high poverty counties compared with low poverty counties. Interestingly, poverty disparities for accidental firearm mortality have been decreasing over time. Time trends by county poverty category along with CIs for each injury mechanism are available in the **S2 Table.**

**Table 2** reports RR for the multivariate negative binomial regressions. Confidence intervals for RR estimates are included in the **S1 Table**. Adjusting for age, race/ethnicity, sex and urbanism, there is a significant and increasing linear association between county poverty and mortality from unintentional injury. Residents in counties with a poverty rate of greater than 20% had a 49% increased risk of death from injury (RR = 1.49; 95% CI 1.33,1.68) compared to residents in low poverty (less than 5%) counties in 1999, compared with a 72% increased risk of death in 2012 (RR 1.72; 95% CI 1.44,2.05).

**Table 2:** Relative Risk of Mortality from Unintentional Injury: United States 1999–2012.

							Specific Cause							
	All Unintentional Injuries		MVA		Falls		Accidental Discharge of Firearms		Drowning		Exposure to Fire/Smoke		Poisoning	
	1999	2012	1999	2012	1999	2012	1999	2012	1999	2012	1999	2012	1999	2012
**Age**														
0-14	0.32**	0.32**	0.19**	0.16**	0.34**	0.2**	0.23**	0.35**	0.99	1.05	1.96**	2.01**	0.07**	0.03**
15-24	1													
25-44	0.95	1.35**	0.68**	0.85**	1.93**	2.05**	0.54**	0.71*	0.67**	0.83*	1.63**	2.17**	3.47**	2.87**
45-64	1.02	1.63**	0.62**	0.79**	5.15**	9.03**	0.37**	0.62**	0.6**	0.94	2.37**	5.24**	2.89**	3.26**
65-84	2.11**	2.57**	0.95	0.96	31.07**	62.34**	0.3**	0.58**	0.78**	0.99	6.62**	10.74**	0.99	0.72**
>85	8.51**	12.11**	1.45**	1.55**	200.66**	429.29**	0.5**	0.31*	1.37*	1.45**	14.19**	21.07**	1.99**	0.7**
**Race/Ethnicity**														
White	1.00	Reference												
Black	1.12**	0.76**	1.09**	0.99	0.73**	0.48**	1.47**	1.45**	1.56**	1.26**	2.76**	1.82**	1.13+	0.58**
Hispanic	0.88**	0.65**	1	0.89**	0.81**	0.66**	0.65**	0.56**	1.16*	0.65**	0.73**	0.59**	0.89+	0.45**
Other	0.88**	0.69**	1.02	0.79**	0.72**	0.65**	0.5**	0.46**	1.27*	1.31**	0.71**	0.68**	0.44**	0.49**
**Sex**														
Female	1.00	Reference												
Male	2.25**	2.05**	2.15**	2.36**	2.47**	1.99**	6.24**	6.68**	4.19**	3.74**	1.83**	1.71**	2.65**	2**
**Urbanism**														
Large Central Metro	1.00	Reference												
Large Fringe Metro	1.23**	1.08+	1.62**	1.44**	0.81**	0.87*	1.58**	1.44+	1.1	1.15+	1.22*	1.23*	0.61**	0.87+
Medium Metro	1.37**	1.25**	1.68**	1.48**	0.92	0.99	1.85**	2.35**	1.31**	1.26**	1.5**	1.42**	0.82**	1.1
Small Metro	1.5**	1.37**	1.86**	1.76**	0.99	0.94	2.07**	3.15**	1.46**	1.23*	1.64**	1.93**	0.61**	0.94
Micropolitan	1.82**	1.62**	2.43**	2.32**	0.92	0.99	3.39**	3.54**	1.55**	1.55**	1.95**	2.14**	0.63**	1.06
Rural	2.34**	1.95**	3.31**	3.14**	0.95	0.99	4.47**	4.18**	1.94**	1.64**	2.3**	2.99**	0.6**	1.03
**County Poverty**														
0-<5%	1.00	Reference												
5-<10%	1.13*	1.27**	1.21**	1.5**	0.89	1.24	1.22	1.27	1.33+	1.61	1.62**	1.79	1.16	1.19
10-<15%	1.33**	1.44**	1.55**	1.76**	0.9	1.23	2.95**	1.77	1.58**	2.08*	2.45**	2.17	1.23	1.51*
15-<20%	1.41**	1.56**	1.7**	2.11**	0.85	1.21	3.23**	2.09	1.53**	2.29*	2.5**	2.73*	1.32*	1.64**
≥20%	1.49**	1.72**	1.79**	2.44**	0.89	1.13	3.76**	3.02	1.52*	2.26*	2.49**	3.65*	1.49**	1.78**

Source: National Center for Health Statistics (Compressed Mortality File 1999–2012

** p <0.01

* p <0.05

+ p<0.10

Relative risk increased from 1999 to 2012 for all mechanisms with the exception of accidental discharge of firearms. RR of death from MVA for counties with >20% poverty increased from 1.79 (95% CI 1.56,2.06) to 2.44 (95% CI 1.90,3.12) between 1999 and 2012. Similar increases were seen for drowning, exposure to smoke/fire and poisonings.

**Table 3** reports the number of deaths per year for each injury mechanism and the corresponding Population Attributable Fraction (PAF) for county poverty. Again, the PAF represents the percentage of deaths that may not have occurred had fatality rates across all counties been equivalent to the counties with the lowest poverty rates. Between 1999 and 2012 over 1.6 million people died from unintentional injuries. The PAF for all unintentional injuries was 0.22 (95% CI 0.13,0.30) in 1999, and rose steadily to 0.35 (95% CI 0.22,0.45) in 2012. The total number of deaths due to unintentional injury was over 127,000 in 2012, meaning that approximately 44,700 (95% CI 28,606–58,093) excess deaths occurred in 2012 associated with county poverty. Over the 14 year period from 1999 to 2012, 488,015 (95% CI 317,553–626,187)deaths from injuries may not have occurred if the risk across all counties was the same as those counties with the lowest poverty rates.

**Table 3:** Injury Fatalities, Population Attributable Fraction, and Attributable Deaths for County Poverty, 1999–2010.

				Specific Cause																	
	All Unintentional Injuries			MVA			Falls^a^			Accidental Discharge of Firearms			Drowning			Exposure to Smoke/Fire			Poison		
	Total		Attributable	Total		Attributable	Total		*Attributable*	Total		Attributable	Total		Attributable	Total		Attributable	Total		Attributable
Year	Deaths	PAF	Deaths	Deaths	PAF	Deaths	Deaths	*PF*^a^	*Deaths*^a^	Deaths	PAF	Deaths	Deaths	PAF	Deaths	Deaths	PAF	Deaths	Deaths	PAF	Deaths
**1999**	97860	0.22	21100	42401	0.31	13300	13162	*0*.*11*	*1421*	824	0.58	482	3529	0.32	1119	3348	0.53	1781	12186	0.18	2252
**2000**	97900	0.16	15476	43354	0.24	10225	13322	*0*.*15*	*2064*	776	0.46	356	3482	0.30	1058	3377	0.46	1550	12757	0.18	2258
**2001**	101537	0.22	22652	43788	0.27	11943	15019	*0*.*17*	*2596*	802	0.62	497	3281	0.34	1113	3309	0.46	1515	14078	0.39	5542
**2002**	106742	0.22	23404	45380	0.27	12160	16257	*0*.*21*	*3380*	762	0.57	438	3447	0.36	1248	3159	0.64	2006	17550	0.34	5978
**2003**	109277	0.23	25089	44757	0.31	13663	17229	*0*.*23*	*3976*	730	0.69	503	3306	0.42	1390	3369	0.71	2377	19457	0.32	6219
**2004**	112012	0.29	32782	44933	0.35	15825	18807	*0*.*12*	*2243*	649	0.35	225	3308	0.32	1063	3229	0.43	1390	20950	0.42	8890
**2005**	117809	0.34	40074	45343	0.37	16990	19656	*0*.*06*	*1250*	789	0.74	581	3582	0.35	1266	3197	0.53	1701	23618	0.50	11786
**2006**	121599	0.35	42350	45316	0.34	15547	20823	*0*.*03*	*673*	642	0.38	244	3579	0.18	657	3109	0.47	1456	27531	0.55	15083
**2007**	123706	0.38	47184	43945	0.49	21668	22631	*0*.*08*	*1783*	613	0.82	504	3443	0.47	1619	3286	0.37	1217	29846	0.41	12172
**2008**	121902	0.35	42104	39790	0.46	18431	24013	*0*.*05*	*1268*	592	0.75	446	3548	0.30	1056	2912	0.47	1366	31116	0.43	13434
**2009**	118021	0.32	37741	36216	0.40	14591	24792	*0*.*12*	*2877*	554	0.58	321	3517	0.49	1735	2756	0.68	1878	31758	0.47	14831
**2010**	120859	0.40	47813	35332	0.40	14176	26009	0.02^b^	520^b^	606	0.53	321	3782	0.79	3002	2782	0.69	1908	33041	0.51	16928
**2011**	126438	0.36	45518	35303	0.51	18005	27483	*0*.*09*	*2473*	591	0.58	343	3556	0.93	3307	2746	0.68	1867	36280	0.46	16689
**2012**	127792	0.35	44727	36415	0.50	18208	28753	0.18^b^	5175^b^	548	0.52	285	3551	0.54	1918	2464	0.62	1528	36332	0.37	13443
**TOTAL**	**1603454**	**0.30**	**488015**	**582273**	**0.37**	**214732**	**287956**	***0*.*12***	***23011***	**9478**	**0.58**	**5546**	**48911**	**0.44**	**21550**	**43043**	**0.55**	**23541**	**346500**	**0.40**	**145505**

Source: National Center for Health Statistics (Compressed Mortality File 1999–2010)

^a^ PAF for falls is negative, and so the Preventative Fraction (PF) is calculated in its place (1-PF = 1/[1-PAF]). The PF is the proportion reduction in the outcome if all individuals were exposed to a protective factor.

^b^ PAF for falls in 2010 and 2012 was positive, so this number is a true PAF with associated attributable deaths

## Comment

In this study, we use comprehensive annual injury mortality data combined with county-level census poverty measures to characterize disparities in injury mortality in the US from 1999–2012. Injury mortality has increased over the past decade, with disparate trends identified for specific injury mechanisms. In accordance with the growing body of literature documenting the deleterious effects of neighborhood poverty on health, our study shows that county-level poverty confers a greater risk of death from unintentional injury, and that higher poverty areas have shouldered the burden of the recent national increases in unintentional injury mortality rates.

There is an increasing socioeconomic disparity for all combined unintentional injuries. These findings are in line with recent research that has found widening area-based disparities for all-cause mortality and life expectancy[26,6]. Unintentional poisoning deaths have been on the rise since at least the 1980s[27], and the largest driver of this trend has been an increase in prescription drug overdose[28]. In 2008, opioid analgesics (including morphine, oxycodone, methadone, and hydrocodone, among others) were responsible for 55% of deaths due to drug overdose[29]. The disparate increase within high poverty and rural areas deserves greater attention and future research. Recent research has raised concerns regarding nonrandom misclassification of unintentional poisonings based on both area and individual sociodemographic characteristics, due in part to wide variations in the qualifications and training of medical examiners[30].

While the PAF increased for nearly all mechanisms, it is important to note that the equation for PAF takes into account both the prevalence of the exposure and the relative risk. County poverty rates have increased over the past decade. The distribution of counties across poverty categories has shifted such that fewer counties fall within the low poverty group. This increase in exposure can increase the PAF without any change in relative risk. Moreover, caution is advised in interpreting the PAF as a causal statement about county poverty[31]. However, the PAF is powerful in reflecting the impact of increases over time in both the exposure of interest (county poverty) and the relative risk.

Despite the use of comprehensive mortality data, there are several limitations. First, although associations between individual socioeconomic status and injury rates have been documented[32,33], information on socioeconomic status of individuals is not available in the CDC dataset. We should not assume that county poverty reflects the SES socioeconomic status of individuals within counties; however, we also cannot conclude that county-level poverty is associated with injury mortality *above and beyond* an individual’s poverty status. What we show as a possible poverty effect may indeed represent a combined effect of individual poverty status and the area poverty[34]. Despite this limitation, the use of area level poverty as a means to determine risk and identify disparity is highly valuable, and rigorous documentation of poverty-based disparities is an important and necessary foundation for future research.

The mechanisms linking area poverty and injury are not explored in this study, and likely vary based on the specific injury outcome. Area poverty may operate through various and interdependent mechanisms that increase injury risk, including decayed physical environments (e.g. deteriorating streets/sidewalks, poor access to safe green space)[35,36], restricted access to health information and safety equipment (e.g. bike helmets, newer/safer cars)[37], and social isolation and norms regarding risky behaviors (e.g. seat belt use, drug use)[38–41]. Once again, future research is needed to identify which aspects of the social and/or physical environment contribute to the increased likelihood of death from injury.

## Conclusion

This study documents area-based socioeconomic disparities in injury mortality across categories of unintentional injury. In contrast to disease mortality, rates of unintentional injuries have risen over the past decade, with the greatest burden shouldered by populations living in high poverty areas. Our findings shed light on the potentially important role of the socioeconomic and physical environment in shaping patterns of injury mortality. Given the trends of *increasing* areal socioeconomic inequality, these results also highlight the importance of community-level intervention. Future research, as well as public health policies aimed at reducing injury rates, should take seriously the neighborhood environments to which individuals are exposed.

## Supporting Information

S1 Table Table 2 Supplement: Relative Risk of Mortality from Unintentional Injury: United States 1999–2012. (XLSX)

S2 Table Fig 1 Supplement: Mortality Trends^a^ by County Poverty Category. (XLSX)

S3 Table Table 3 Supplement: Injury Fatalities, Population Attributable Fraction, and Attributable Deaths for County Poverty, 1999–2010. (XLSX)

## References

[1] Heron M. Deaths: Leading causes for 2010. National Vital Statistics Reports 2013; 62(6) 24364902

[2] Murphy SL, Xu J, Kochanek KD. Deaths: Final Data for 2010. National Vital Statistics Reports 2013; 61(4) 24979972

[3] Rockett IR, Regier MD, Kapusta ND, Coben JH, Miller TR, Hanzlick RL, et al Leading causes of unintentional and intentional injury mortality: United States, 2000–2009. American Journal of Public Health 2012; 102(11): 84–92 10.2105/AJPH.2012.300960 PMC3477930 22994256

[4] Hu G, Baker SP. Trends in unintentional injury deaths, U.S., 1999–2005: age, gender and racial/ethnic differences. American Journal of Preventive Medicine 2009; 37(3): 188–194 10.1016/j.amepre.2009.04.023 19595555

[5] Paulozzi LJ, Ballesteros MF, Stevens JA. Recent trends in mortality from unintentional injury in the United States. Journal of Safety Research 2006; 37(3): 277–283 16828115 10.1016/j.jsr.2006.02.004

[6] Singh GK, Siahpush M. Widening socioeconomic inequalities in US life expectancy, 1980–2000, International Journal of Epidemiology. 2006; 35(4): 969–979 16684899 10.1093/ije/dyl083

[7] Chen JT, Rehkopf DH, Waterman PD, Subramanian SV, Coull BA, Cohen B, et al Mapping and measuring social disparities in premature mortality: The impact of census tract poverty within and across Boston neighborhoods, 1991–2001, Journal of Urban Health 2006; 83(6): 1063–1084 10.1007/s11524-006-9089-7 PMC3261292 17001522

[8] Diez Roux AV, Merkin SS, Arnett D, Chambless L, Massing M, Nieto FJ, et al Neighborhood of Residence and Incidence of Coronary Heart Disease. New England Journal of Medicine 2001; 345:99–106. 11450679 10.1056/NEJM200107123450205

[9] Morenoff JD. Neighborhood Mechanisms and the Spatial Dynamics of Birth Weight. American Journal of Sociology 2003; 108(5): 976–1017 10.1086/374405 14560732

[10] Beck AF, Moncrief T, Huang B, Simmons JM, Sauers H, Chen C, et al Inequalities in neighborhood child asthma admission rates and underlying community characteristics in one US county. Journal of Pediatrics 2013; 163(2): 574–80 10.1016/j.jpeds.2013.01.064 23522864 PMC3746008

[11] Ludwig J, Sanbonmatsu L, Gennetian L, Adam E, Duncan GJ, Katz LF, et al Neighborhoods, obesity and diabetes—A randomized social experiment. New England Journal of Medicine 2011; 365: 1509–1519 10.1056/NEJMsa1103216 22010917 PMC3410541

[12] Diez Roux AV, Mair C. Neighborhoods and health. Annals of the New York Academy of Science 2010; 1186: 125–145 10.1111/j.1749-6632.2009.05333.x 20201871

[13] Sampson RJ, Morenoff JD, Gannon-Rowley T. Assessing ‘neighborhood effects’: Social processes and new directions in research. Annual Review of Sociology; 2001: 28: 443–478

[14] Bishaw A. Poverty: 2000–2012. American Community Survey Briefs 2013.

[15] Burrows S, Auger N, Gamache P, Hamel D. Individual and area socioeconomic inequalities in cause-specific unintentional injury mortality: 11-year follow-up study of 2.7 million Canadians. Accident Analysis and Prevention 2012; 45: 99–106 10.1016/j.aap.2011.11.010 22269490

[16] Birken CS, Parkin PC, To T, Macarthur C. Trends in rates of death from unintentional injury among Canadian children in urban areas: influence of socioeconomic status, CMAJ 2006; 175(8): 867–868 16998078 10.1503/cmaj.051207 PMC1586087

[17] Singh GK, Azuine RE, Siahpush M, Kogan MD. All-cause and cause-specific mortality among US youth: Socioeconomic and rural-urban disparities and international patterns. Journal of Urban Health 2012; 90(3): 388–405 10.1007/s11524-012-9744-0 PMC3665977 22772771

[18] Cubbin C, LeClere FB, Smith GS. Socioeconomic status and injury mortality: Individual and neighborhood determinants. Journal of Epidemiology and Community Health 2000, 54: 517–534 10846194 10.1136/jech.54.7.517 PMC1731715

[19] Zarzaur BL, Croce MA, Fabian T, Fischer P, Magnotti LJ. A population-based analysis of neighborhood socioeconomic status and injury admission rates and in-hospital mortality. Journal of the American College of Surgeons 2010; 211: 216–223 10.1016/j.jamcollsurg.2010.03.036 20670859 PMC3042251

[20] Ali MT, Hui X, Hashmi ZG, Dhiman N, Scott VK, Efron DT, et al Socioeconomic disparity in inpatient mortality after traumatic injury in adults. Surgery 2013; 154(3): 461–467 10.1016/j.surg.2013.05.036 23972652 PMC3989530

[21] National Center for Health Statistics. Compressed Mortality File, 1999–2010 (machine readable data file and documentation, CDROM Series 20, No. 2P) as compiled from data provided by the 57 vital statistics jurisdictions through the Vital Statistics Cooperative Program. Hyattsville, Maryland. 2012.

[22] U.S. Census website. Available: http://www.census.gov/hhes/www/poverty/methods/definitions.html.

[23] Krieger N, Chen JT, Waterman PD, Rehkopf DH, Subramanian SV. Painting a truer picture of US socioeconomic and racial/ethnic health inequalities: The Public Health Disparities Geocoding Project. Am J Public Health 2005; 95: 312–323. 15671470 10.2105/AJPH.2003.032482 PMC1449172

[24] Bains N. Standardization of rates, 2009. Available: http://www.apheo.ca/resources/indicators/Standardization%20report_NamBains_FINALMarch16.pdf. Accessed 2014 Jan 14.

[25] Tanuseputro P, Manuel DG, Schultz SE, Johansen H, Mustard CA. Improving Population Attributable Fraction Methods: Examining Smoking-attributable Mortality for 87 Geographic Regions in Canada. American Journal of Epidemiology 2005; 161(8): 787–798. 15800272 10.1093/aje/kwi093

[26] Daly LE. Confidence limits made easy: interval estimation using a substitution method. Am J Epidemiol 1998;147:783–90. 9554420 10.1093/oxfordjournals.aje.a009523

[27] Singh GK. Area deprivation and widening inequalities in US mortality, 1969–1998. American Journal of Public Health 2003; 93(7): 1137–1143 10.2105/ajph.93.7.1137 PMC1447923 12835199

[28] Fingerhut LA, Cox CS. Poisoning mortality, 1985–1995. Public Health Reports 1998; 113(3): 218–233. 9633866 PMC1308672

[29] Centers for Disease Control and Prevention. Vital signs: overdoses of prescription opioid pain relievers—United States, 1999–2008. MMWR Morbidity and Mortality Weekly Report 2011; 60(43): 1487–1492. 22048730

[30] Rockett IR, Wang S, Stack S, De Leo D, Frost JL, Ducatman AM, et al Race/ethnicity and potential suicide misclassification: window on a minority suicide paradox? BMC Psychiatry 2010; 10: 35 10.1186/1471-244X-10-35 20482844 PMC2891687

[31] Durkin MS, Davidson LL, Kuhn L, O’Connor P, Barlow B. Low-income neighborhoods and the risk of severe pediatric injury: a small-area analysis in northern Manhattan. American Journal of Public Health 1994; 84(4): 587–592 8154561 10.2105/ajph.84.4.587 PMC1614793

[32] Levine B. What does the population attributable fraction mean? Preventing Chronic Disease 2007; 4(1) PMC1832135 17173722

[33] Denney JT, He M. The social side of accidental death. Social Science Research 2014; 43: 92–107 10.1016/j.ssresearch.2013.09.004 24267755

[34] Hussey JM. The effects of race, socioeconomic status and household structure on injury mortality in children and young adults. Maternal and Child Health 1997; 10.1023/a:1022318610868 10728247

[35] Subramanian SV, Chen JT, Rehkopf DH, Waterman PD, Krieger N. Comparing individual- and area-based socioeconomic measures for the surveillance of health disparities: A multilevel analysis of Massachusetts births, 1989–1991. American Journal of Epidemiology 2006; 164(9):823–34 16968866 10.1093/aje/kwj313

[36] Kelly CM, Schootman M, Baker E, Barnidge EK, Lemes A. The association of sidewalk walkability and physical disorder with area-level race and poverty. Journal of Epidemiology and Community Health 2007; 61: 978–983 17933956 10.1136/jech.2006.054775 PMC2465610

[37] Wen M, Zhang X, Harris CD, Holt JB, Croft JB. Spatial disparities in the distribution of parks and green spaces in the USA. Annals of Behavioral Medicine 2013; 45(1): 18–27 10.1007/s12160-012-9426-x PMC3590901 23334758

[38] White HL, Low H, Macpherson AK. Neighborhood sociocultural demographics and their association with helmet use in children. Canadian Journal of Epidemiology and Biostatistics 2011; 1(6): 44–50

[39] Chu M. Characteristics of persons who seldom or never wear seat belts, 2002 Statistical Brief #62. 12 2004 Agency for Healthcare Research and Quality, Rockville, MD Available: http://meps.ahrq.gov/mepsweb/data_files/publications/st62/stat62.pdf.

[40] Williams CT, Latkin CA. Neighborhood socioeconomic status, personal network attributes, and use of heroin and cocaine. American Journal of Preventive Medicine 2007; 32(6): S203–10 17543712 10.1016/j.amepre.2007.02.006 PMC1986754

[41] Boardman JD, Finch BK, Ellison CG, Williams DR, Jackson JS. Neighborhood disadvantage, stress and drug use among adults. Journal of Health and Social Behavior 2001; 42(2): 151–165 11467250

